# Headache as the sole presentation of cerebral venous thrombosis: a prospective study

**DOI:** 10.1007/s10194-012-0456-3

**Published:** 2012-05-17

**Authors:** Ângela Timóteo, Nuno Inácio, Sara Machado, Ana Amélia Pinto, Elsa Parreira

**Affiliations:** Department of Neurology, Hospital Prof. Dr. Fernando Fonseca, EPE, IC-19, 2720 Amadora, Portugal

**Keywords:** Headache, Sinus thrombosis, Lateral sinus, Cerebral veins

## Abstract

Headache is the most frequent presenting symptom of cerebral venous thrombosis (CVT), most commonly associated with other manifestations. It has been described as its only clinical presentation in 15 % of patients. There is no typical pattern of headache in CVT. The objective of this study was to study the characteristics of headache as the sole manifestation of CVT. From a prospective study of 30 consecutive patients diagnosed with CVT over 18 months, we selected those who presented with headache only: they had a normal neurological examination, no papilloedema and no blood or any parenchymal lesion on CT scan. All were submitted to a systematic etiological workup and a structured questionnaire about the characteristics of headache was provided. Headache was the sole manifestation of CVT in 12 patients; it was diffuse or bilateral in the majority. Seven patients referred worsening with sleep/lying down, Valsalva maneuvers or straining. There was no association between the characteristics of headache and extension of CVT. Time from onset to diagnosis was significantly delayed in these patients presenting only with headache. In our series, 40 % of patients presented only with headache. There was no uniform pattern of headache apart from being bilateral. There was a significant delay of diagnosis in these patients. Some characteristics of headache should raise the suspicion of CVT: recent persistent headache, thunderclap headache or pain worsening with straining, sleep/lying down or Valsalva maneuvers even in the absence of papilloedema or focal signs.

## Introduction and aims

Cerebral venous thrombosis (CVT) presents with a wide spectrum of manifestations. The most common presenting symptom is headache, frequently associated with other symptoms and signs, namely seizures, focal neurological deficits, papilloedema and impaired consciousness [1]. The clinical picture is determined by the age of patient, site of CVT and the presence or absence of parenchymal lesions [2].

Isolated headache, as the only presentation of CVT, has been described but it is rare [1, 3, 4]. In these circumstances, usually computed tomography (CT) scan and/or cerebrospinal fluid (CSF) examination disclose conditions that explain the headache, such as subarachnoid hemorrhage (SAH), parenchymal lesions or meningitis (this last one frequently related to an infections cause of CVT). Moreover, a typical pattern of headache has not been yet identified [5].

A study from a French group identified 17 patients out of 123 in whom headache was the only manifestation of CVT in the absence of intracranial hypertension (no papilloedema and/or normal CSF pressure), SAH, meningitis or any parenchymal lesion [4]. Another study reported 28 patients with isolated headache out of 62 patients with lateral sinus thrombosis [6].

Our purpose was to study the characteristics of headache as the sole presentation of CVT, namely if there was a typical pattern of headache or if there was an association between its features and the sinuses involved and extension of CVT.

## Methods and patients

From a prospective study of 30 consecutive patients diagnosed with CVT over 18 months, we selected those who presented with headache only. Diagnosis of CVT was made by magnetic resonance imaging (MRI) combined with MR venography (MRV): both increased signal on MRI T1 and T2 weighted images and the absence of flow was required to confirm diagnosis. The presence of parenchymal lesions was assessed on both non-contrast computed tomography scan (NCCT scan) and MRI (T1, T2, fluid-attenuated inversion recovery, diffusion, T2*SW; MRV: coronal 2D TOF). In those cases in which there were initial diagnostic doubts, a lumbar puncture was also performed; in all patients CSF pressure and examination were normal.

Patients were included if they had only headache at presentation and throughout the course of the disease. This is to say that they all had a normal neurological examination, no papilloedema on fundoscopic examination and no blood or any parenchymal lesion on NCCT scan and MRI. All were submitted to a systematic etiological workup in order to identify the causes and risk factors for CVT, such as malignancies, hematological disorders, antiphospholipid syndrome and infectious diseases.

A standardized questionnaire concerning the characteristics of headache (mode of onset, location, severity, pattern and aggravating factors) was applied to all patients by the authors on the day diagnosis was confirmed.

Severity was assessed in three grades (1, 2 and 3). Three modes of onset of headache were defined: “thunderclap” (sudden onset, reaching maximum intensity within 1 min), acute (developing in <24 h) and progressive over 24 h. Headache was classified into different types based on the criteria of the International Headache Society (IHS-2004). All patients were asked about previous history of the headache.

### Statistical analysis

All variables regarding clinical and imaging CVT characteristics were compared using a univariate analysis (χ^2^ test). The level of significance was set at 95 % (*P* = 0.05).

## Results

During the inclusion period, a total of 30 patients were diagnosed with CVT. Twelve patients (40 %) had headache as the only presenting symptom (data summarized in Table 1). The majority of these were young (mean age 39.9 years old), female (91.7 %) patients, with only one-third having a past medical history of headache (in all compatible with the diagnosis of migraine without aura). Although no uniform headache pattern was found, in the majority of the patients the headache was bilateral (91.7 %) even when CVT was unilateral (as in such cases of lateral sinus thrombosis). Moreover, the headache was more frequently severe, tightening in quality and progressive over time (10/12; 83.3 % in each case). In all patients at least one headache associated symptom was identified; in about 58 % of the patients, besides headache, nausea and vomiting were reported. The same frequency of patients complaining of headache worsening with Valsalva maneuvers, sleep/lying down (either waking up in the morning with a more severe headache or waking up in the middle of the night because of the pain) or straining was found.

**Table 1:** Characteristics of patients with isolated headache as manifestation of CVT

Patient	Gender	Age	Previous history of headache	Characteristics of CVT headache				
				Evolution	Location	Pattern	Severity	Associated symptoms
1	M	46	No	Progressive	Bilateral fronto-orbital	Tightening	3	Nausea/vomiting/photophobia
2	F	42	No	Thunderclap	Diffuse	Tightening	2	Kinesiophobia
3	F	41	No	Progressive	Bilateral posterior	Tightening	3	Nausea/vomiting/photophobia/worse Valsalva maneuvers
4	F	46	Migraine	Progressive	Bilateral posterior	Throbbing	3	Nausea/vomiting/kinesiophobia
5	F	29	No	Progressive	Bilateral fronto-orbital	Tightening	3	Nausea/vomiting/worse straining
6	F	17	No	Progressive	Bilateral fronto-orbital	Tightening	3	Nausea/vomiting/worse straining
7	F	38	No	Progressive	Left hemicranium	Tightening	3	Nausea/vomiting/worse Valsalva maneuvers
8	F	38	No	Progressive	Diffuse	Tightening	2	Worsening with sleep/lying down
9	F	68	Migraine	Progressive	Bilateral posterior	Tightening	3	No
10	F	40	Migraine	Progressive	Bilateral posterior	Tightening	3	Worsening with sleep/lying down
11	F	48	Migraine	Thunderclap	Diffuse	Throbbing	3	Worse Valsalva maneuvers
12	F	26	No	Progressive	Diffuse	Tightening	3	Nausea/vomiting/photophobia

The authors tried to establish a relationship between the affected sinus and the characteristics of headache, but no significant association was found. With regard to the etiology of CVT, one patient had CVT during puerperium. All other patients had no predisposing factor besides oral contraceptive use.

The lateral sinus was involved in more than half of the patients (either alone or in association with jugular vein or with other sinuses). In these cases, about 87.5 % of the patients’ first NCCT scan had signs suggesting CVT (Table 2).

**Table 2:** Particulars of diagnosis in CVT patients with isolated headache

Patient	Time from onset to diagnosis (days)	Previous evaluations	NCCT scan	Site of thrombosis
1	7	1	Spontaneous hyperdensity	LLS, LJV
2	13	–	Normal	Anterior ½ SSS
3	5	1	Spontaneous hyperdensity	T, LLS, RLS
4	15	–	Normal	SSS
5	21	2	Normal	T
6	21	1	Spontaneous hyperdensity	RLS
7	10	–	Spontaneous hyperdensity	LLS
8	3	–	Normal	LLS
9	2	–	Normal	RLS, RLV
10	10	–	Normal	SSS
11	1	–	Spontaneous hyperdensity	LLS
12	12	1	Spontaneous hyperdensity	LLS, LJV

*LLS* left lateral sinus, *LJV* left jugular vein, *SSS* superior sagittal sinus, *RLS* right lateral sinus, *RJV* right jugular vein, *T* torcule

Five patients had already been evaluated previously in the emergency room and had been discharged diagnosed with primary headache (Table 2).

We did an additional analysis comparing the time needed until the final diagnosis of CVT reached in patients with isolated headache and all others presenting with distinct manifestations (data regarding these patients not shown). We found out that the diagnosis was significantly delayed in those patients with isolated headache—9 ± 6.7 days in patients with isolated headache versus 4.5 ± 4.2 days in all other patients, *P* < 0.05.

Despite the significantly delayed diagnosis in this group, all patients had a favorable outcome: none developed complications and, in the majority, headache resolved within 1 month.

## Discussion and conclusions

In our prospective series of patients diagnosed with CVT, 40 % presented with headache only. There was no uniform pattern of headache apart from being bilateral: the majority of the patients presented with a diffuse or anterior/posterior bilateral headache, in contrast to what was found in other series of CVT patients, in which headache was more frequently characterized as being unilateral [4, 9]. The headache was more frequently severe, tightening in quality and progressive. Two patients presented with a thunderclap headache: one of them had a spontaneous hyperdensity on NCCT scan but the other one had a completely normal NCCT scan, a normal CSF pressure and a normal CSF examination. This strengthens the need for performing a brain MRI combined with an MRV in these patients presenting with thunderclap headache.

Nausea and/or vomiting were also frequent features. Aggravating factors such as worsening with sleep/lying down, Valsalva maneuvers and straining were present in seven patients.

Unilateral lateral sinus thrombosis, either alone or associated with jugular vein occlusion, was seen in seven patients; this tendency of lateral sinus thrombosis to present solely with headache was observed in other studies [3, 4, 7]. Nonetheless, we had not found a statistically significant association between characteristics of headache or associated symptoms and signs (in those patients from the initial series of CVT that presented with a distinct clinical picture) and extension of CVT or involved sinuses. One of our patients had bilateral involvement of lateral sinus, frequently related to intracranial hypertension [4]; other two had superior sagittal sinus thrombosis, classically associated with motor deficits and seizures [1], which our patients did not present with.

Time from onset to diagnosis was significantly delayed in these patients presenting only with headache, when comparing to those that presented with other symptoms and signs. Nearly half of the patients had already been evaluated previously by a physician unfamiliarized with CVT in the emergency room and had been discharged diagnosed with migraine or tension-type headache. This fact points to a need for making a detailed history of headache and performing an MRI/MRV in patients presenting with a recent persistent headache.

The mechanism of headache in these patients who have no intracranial hypertension, SAH, meningitis or parenchymal lesions remains unexplained [1]. A local inflammatory reaction determining dilation of vessels in the walls of the affected sinuses is a possibility, and extravasation of inflammatory proteins could explain the bilateral pain. The pain may also be caused by the stretching of nerve fibers in the walls of the occluded sinus [8].

It can be said that these 12 patients had a favorable prognosis, as they presented only with headache and did not have any complication over the course of the disease.

The most important finding of this study was that isolated headache as presenting manifestation of CVT is much more frequent than what has been pointed out previously in other studies. This stresses the idea to systematically look for CVT in patients with recent persistent headache, thunderclap headache or pain worsening with straining, sleep or Valsalva maneuvers, even in the absence of papilloedema or focal signs.
